# Targeted next generation sequencing of well-differentiated/dedifferentiated liposarcoma reveals novel gene amplifications and mutations

**DOI:** 10.18632/oncotarget.24924

**Published:** 2018-04-13

**Authors:** Neeta Somaiah, Hannah C Beird, Andrea Barbo, Juhee Song, Kenna R. Mills Shaw, Wei-Lien Wang, Karina Eterovic, Ken Chen, Alexander Lazar, Anthony P. Conley, Vinod Ravi, Patrick Hwu, Andrew Futreal, George Simon, Funda Meric-Bernstam, David Hong

**Affiliations:** ^1^ Department of Sarcoma Medical Oncology, Division of Cancer Medicine, University of Texas MD Anderson Cancer Center, Houston 77030, TX, USA; ^2^ Department of Genomic Medicine, University of Texas MD Anderson Cancer Center, Houston 77030, TX, USA; ^3^ Department of Biostatistics, University of Texas MD Anderson Cancer Center, Houston 77030, TX, USA; ^4^ Khalifa Institute for Personalized Cancer Therapy (IPCT), University of Texas MD Anderson Cancer Center, Houston 77030, TX, USA; ^5^ Department of Pathology, University of Texas MD Anderson Cancer Center, Houston 77030, TX, USA; ^6^ Division Chair, Division of Cancer Medicine, University of Texas MD Anderson Cancer Center, Houston 77030, TX, USA; ^7^ Department of Investigational Cancer Therapeutics, University of Texas MD Anderson Cancer Center, Houston 77030, TX, USA; ^8^ Department of Thoracic Medical Oncology, University of Texas MD Anderson Cancer Center, Houston 77030, TX, USA

**Keywords:** well-differentiated and dedifferentiated liposarcoma sequencing, targeted therapy, MDM2 inhibitors

## Abstract

Well-differentiated/dedifferentiated liposarcoma is a common soft tissue sarcoma with approximately 1500 new cases per year. Surgery is the mainstay of treatment but recurrences are frequent and systemic options are limited. ‘Tumor genotyping’ is becoming more common in clinical practice as it offers the hope of personalized targeted therapy. We wanted to evaluate the results and the clinical utility of available next-generation sequencing panels in WD/DD liposarcoma. Patients who had their tumor sequenced by either FoundationOne (*n* = 13) or the institutional T200/T200.1 panels (*n* = 7) were included in this study. Significant copy number alterations were identified, but mutations were infrequent. Out of the 27 mutations detected in 7 samples, 8 (*CTNNB1, MECOM, ZNF536, EGFR, EML4, CSMD3, PBRM1, PPP1R3A*) were identified as deleterious (on Condel, PolyPhen and SIFT) and a truncating mutation was found in *NF2*. Of these, *EGFR* and *NF2* are potential driver mutations and have not been reported previously in liposarcoma. *MDM2* and *CDK4* amplification was universally present in all the tested samples and multiple other recurrent genes with high amplification or high deletion were detected. Many of these targets are potentially actionable. Eight patients went on to receive an MDM2 inhibitor with a median time to progression of 23 months (95% CI: 10-83 months).

## INTRODUCTION

Liposarcoma is the most common type of soft tissue sarcoma in adults and consists of three distinct subtypes. The well-differentiated (WD)/dedifferentiated/(DD) subgroup is the most common with an incidence of approximately 1000 new cases per year, followed by myxoid/round cell and pleomorphic subtypes [1]. Distinguishing one liposarcoma subtype from another can be challenging at times and histologic examination by an experienced soft tissue sarcoma pathologist is best supplemented with molecular studies for accurate diagnosis. Molecular studies have occasionally resulted in reclassification of incorrectly diagnosed cases [2, 3]. An accurate diagnosis is critical since the disease course and response to treatment varies between subtypes and we might increasingly see more subtype specific clinical trials in the future.

WD and DD liposarcomas universally exhibit amplification of chromosome 12q13-15. Consequently, *MDM2* (mouse double minute 2 homolog, an inhibitor of the tumor suppressor gene *p53*) and *CDK4* (cyclin-dependent kinase 4, a critical regulator of cell cyclin), two well-known oncogenes are also amplified. By histology, WD liposarcomas are characterized by the presence of adipocytes of varying sizes with prominent fibrous stroma (lipoma-like, sclerosing, and inflammatory variants have been described). DD liposarcomas, on the other hand, typically have a highly cellular, spindle cell-rich DD portion with 5 or more mitoses per 10 high power fields (hpfs) in conjunction with an adipocyte-rich, WD portion [1]. DD histology has been associated with much more aggressive clinical course and poorer outcomes [4, 5]. The exact clonal relationship between WD and DD liposarcoma is not clear; about 25–40% of patients with WD will manifest DD histology at recurrence, but the reverse transformation is seen as well [4].

The most common site of origin for a WD or DD liposarcoma is the retroperitoneum, but these tumors can also arise in the extremities, paratesticular areas, or the trunk. These tumors can be massive in size at diagnosis (frequently >30 cm in the retroperitoneum) and invade adjacent viscera and structures. There are no known risk factors for the development of this disease and no specific gender or age predilection with a median age of around 61 yrs. These tumors carry a very high rate of local recurrence and locoregional morbidity, but distant metastasis is not very common. WD liposarcoma will rarely metastasize, whereas DD liposarcoma has a 10-20% risk for distant metastasis, typically to the lungs [6]. Surgery is the mainstay of treatment and patients often undergo multiple re-operations with increasing surgical morbidity. WD liposarcoma is largely resistant to conventional cytotoxic chemotherapy and radiation therapy [7], and as a result, treatment options other than surgery, are limited. Italiano *et al.* reported a multicenter, retrospective study of 208 WD and DD liposarcoma patients, 82% of which were treated with an anthracycline-containing regimen. The ORR was only 12% and all of the responses occurred in anthracycline treated patients. Rates of 3- and 6 month PFS were 59% and 44% [8]. Review of the MD Anderson Cancer Center (MDACC) experience revealed a higher response rate in DD liposarcoma with a RECIST (Response Evaluation Criteria In Solid Tumors) response rate to first-line chemotherapy of 22% and this is likely due to the more frequent use of doxorubicin plus ifosfamide in combination compared to single agent therapy [9].

In the past decade, a better understanding of the distinct genetic and molecular aberrations has not only helped with more accurate diagnosis but has also made available novel targeted therapy options (i.e. MDM2 inhibitors and CDK4 inhibitors). Current technology has made next generation sequencing on FFPE samples using gene panels a reliable method to detect amplifications and deletions [10]. ‘Tumor genotyping’ is becoming more common in clinical practice as it offers the hope of personalized targeted therapy and identifying novel targets on the tumor. Herein we report next-generation sequencing results for WD/DD liposarcoma patients and the clinical utility of such an approach using currently available genotyping panels.

## RESULTS

### Patients Characteristics

We identified 20 patients with advanced, relapsed WD/DD liposarcoma whose tumors had been sent for molecular profiling (Table 1). Thirteen (65%) of the samples were processed through Foundation Medicine, Cambridge, MA and 7 (35%) through the MD Anderson Institute of Personalized Medicine, Houston, TX (five out of the 7 were analyzed on T200 and 2 on T200.1 platform). The median age of this group at the time of diagnosis was 50 years (range: 31–77 years). Thirteen (65%) patients had a diagnosis of DD liposarcoma at some point during their disease course and out of the 20 samples that were sent for sequencing, 11 (55%) were DD and 9 (45%) were WD liposarcoma, with 3 of the WD samples having hypercellular areas (cellular WD liposarcoma). All samples analyzed were obtained at the time of surgery. Median number of resections for liposarcoma was 3 (range:1–5). Majority of samples sent for sequencing were systemic therapy naïve, with only 3 (15%) having had treatment prior to sample collection.

**Table 1:** Characteristics of 20 patients with WD/DD liposarcoma who underwent targeted next-generation sequencing

Characteristics	*n* (%)
Gender	
Male	11 (55%)
Female	9 (45%)
Age at diagnosis, years	
Median	50
Range	31–77
Location of Liposarcoma	
Neck	2 (10%)
RP	17 (85%)
Pelvic	1 (5%)
Number of surgeries	
Median	3
Range	1–5
Histology Sequenced	
DDL	11 (55%)
Conventional WD Liposarcoma	6 (30%)
Cellular WD Liposarcoma	3 (15%)

The median follow-up time for all patients was eight years (95% Confidence Interval (CI): 5–11 years). Eight (40%) patients died within the study period and the median OS time from the time of initial diagnosis was 10 years (95% CI: 5 years–not reached) (Supplementary Figure 1). About 5% of the patients have died by the 2nd year, 23% by the 5th year and 50% by the 10th year. There was not sufficient evidence to conclude that pathology (DDL versus WDL), or sequencing technology was associated with OS among all patients (*p* = 0.55 and 0.28, respectively) (Supplementary Figure 2).

Significant CNAs (high amplifications/high deletions) were identified in all 20 samples. Only recurrent (present in ≥2 samples) CNAs (out of the 166 identified) are listed in Table 2. *MDM2* and *CDK4* amplification were the only two overlapping gene amplifications identified that were universally present in all the samples that were tested for these genes. All patients were p53 wild-type. In the T200/T200.1 panels, 27 mutations were detected in the 7 patients, out of which 8 genes (*CTNNB1, MECOM, ZNF536, EGFR, EML4, CSMD3, PBRM1, PPP1R3A*) were identified as deleterious (on Condel, PolyPhen and SIFT) and a truncating mutation was found in *NF2* (Supplementary Table 2). Of these, only *EGFR* (missense mutation; R108K aGa/aAa) and *NF2* (nonsense mutation; K20* Aag/Tag) are driver mutations and the *EGFR* mutation is potentially actionable. These mutations have not been reported previously in DD liposarcoma. On the FM panel, *ZNF536, CSMD3 and PPP1R3A* were not evaluated (NE) and no mutations were reported in the 13 samples. One sample on the FM panel had a GAPDH-BCL6 fusion.

**Table 2:** Recurrent Copy Number Alterations by sequencing platform

Gene	Gene locus	T200/T200.1 *N* = 7	FM *N* = 13	Previously reported amplifications in DD LPS (55 samples in cbioportal.org)^**^ N (%)	Available targeted agents (with approved indications in other cancers or in clinical testing)
High Amplification (≥4)					
MDM2	12q14.3-q15	4^*^^	13	44 (80%)	MDM2 inhibitors or Nutlins that inhibit MDM2-p53 interaction.
CDK4	12q14	7	13	41 (75%)	CDK 4/6 inhibitors
LRP1	12q13.3	2	0	8 (15%)	NA
NOTCH1	9q34.3	3	0	4 (7%)	Gamma Secretase inhibitors (GSIs)
NOTCH4	6p21.3	2	NE	0	Gamma Secretase inhibitors (GSIs)
DDR1	6p21.3	2	NE	0	DDR1 inhibitor
DAXX	6p21.32	2	0	0	NA
AURKB	17p13.1	2	0	2 (4%)	AURKB inhibitors
ERBB2	17q12	2	0	0	HER2 inhibitors, monoclonal antibodies, and targeted vaccines
MAP2K4	17p12	2	0	1 (2%)	JNK1 inhibitor
FLT4	5q35.3	2	0	4 (7%)	FLT4 inhibitors
FGFR4	5q35.2	2	0	4 (7%)	FGFR4 inhibitors
GATA1	Xp11.23	2	0	3 (6%)	NA
BCL2	18q21.33	1^*^	1	0	BCL2 inhibitor and potential resistance to mTOR inhibitors
NFKB2	10q24.32	2^*^	NE	0	NA
MEN1	11q13.1	2	0	1 (2%)	NA
AKT1	14q32.32	2	1	1 (2%)	AKT or mTOR inhibitors
MCL1	1q21	0^*^	2	2 (4%)	NA
High Deletion					
BRCA2	13q13.1	2	0	0	PARP inhibitors
FLT3	13q12.2	3	0	1 (2%)	NA
FLT1	13q12.3	2	0	1 (2%)	NA
JAK2	9p24.1	2	0	1 (2%)	NA

NE = not evaluated; NA = not yet available

^*^These genes were only included on T200.1 hence analyzed in 2 patient samples.

^^^2 additional samples (in addition to those positive on T200.1) were sent for *MDM2* amplification by FISH for diagnosis confirmation and were found to be positive.

^**^DD liposarcoma samples available through cbioportal.org (includes the samples from study by Barretina *et al.* [15])

Eighteen patients (90%) received systemic therapy (chemotherapy or targeted therapy) at some point in their treatment course. Interestingly, eight patients (two DD liposarcoma, one cellular WD liposarcoma and five with conventional WD liposarcoma) received an MDM2 inhibitor on a phase I clinical trial based on the molecular sequencing data. The median duration of therapy was 11 months (Range <1–80 months). Median time to progression among these patients who received an MDM2 inhibitor was 23 months (95% CI: 10–83 months). Seven of them demonstrated disease stabilization or shrinkage (but not meeting criteria for RECIST partial response). One patient discontinued therapy after 12 days, prior to disease assessment due to declining performance status and three others discontinued (after three months, five months and one year, respectively) due to toxicity (prolonged thrombocytopenia) and not for disease progression.

## DISCUSSION

Our study is the first study to describe the findings of targeted next-generation sequencing in WD/DD liposarcoma patients done as part of their clinical management. It is known that virtually all WD and DD liposarcomas have *MDM2* amplification and close to 90% have *CDK4* amplification and this is felt to be an early event in the pathogenesis of these tumors [1, 11, 12]. We have shown that the currently available targeted tumor genotyping panels can reliably detect these molecular amplifications in *MDM2* and *CDK4*. This is useful as it provides a potential therapeutic target; MDM2 and CDK4 inhibitors are being tested in various clinical trials. The molecular profile can also help confirm the diagnosis of WD/DD liposarcoma in those cases where histopathology is not enough to provide an accurate subtype classification and FISH for *MDM2* is not available. These markers are however, not unique to WD and DD liposarcoma and hence this information needs to be used in conjunction with expert histopathologic diagnosis and the clinical history in order to confirm the diagnosis.

The targeted genotyping panels (T200/T200.1 and FM) detected several other CNAs, in addition to *MDM2* and *CDK4*, and many of them were found in more than one tumor sample (Table 2). Though their frequency was lower, it is likely that they are involved in the evolution of a specific dedifferentiation process and could be exploited as therapeutic targets in those cases. The mutation rate was low and no recurrent mutations were found in the 20 patient samples. *EGFR* (R108K) and NF2 (K20*) were the two possible driver mutations that were detected in 2 separate samples and have not been described before in WD/DD liposarcoma. *EGFR* (R108K) is a missense mutation in exon 1-2 of the epidermal growth factor binding and not in the tyrosine kinase domain. The presence of this mutation has been previously described in approximately 14% of malignant glioblastomas. *EGFR* missense mutations have shown to be oncogenic and can be inhibited by EGFR tyrosine kinase inhibitors, such as erlotinib [13, 14]. No much is known about the NF2 (K20*) mutation in cancer patients. In patients with neurofibromatosis type 2, who have germline *NF2* mutations and are predisposed to forming schwannomas, meningiomas, gliomas or neurofibromas; truncating mutations have been associated with a poorer prognosis [15]. In our study we also noted deleterious mutations in 7 other genes (*CTNNB1* (R151H)*, MECOM* (R208C)*, ZNF536* (T688M)*, EML4* (W729L)*, CSMD3* (P627S)*, PBRM1* (N639K)*, PPP1R3A* (C788Y)) that have also not been described previously in WD/DD liposarcoma. The FM panel did not report any mutations in the 13 samples analyzed. The fact that the FM panel does not have normal tissue to verify the somatic nature of calls might restrict their ability from being able to call at a subclonal level. In a previous large-scale genomic sequencing study of soft tissue sarcoma specimens, that included 50 DD liposarcomas, a different *CTNNB1* (T41I) mutation was described in a DD liposarcoma, including 3 other nonrecurring mutations (*CDH1* (N238D), *EPHA1* (A2127) and *FBXW7* (E113fs)) [16].

Other studies using whole exome and targeted sequencing have also revealed a similar high amplification/ deletion rate but a low frequency of recurrent mutations in the WD/DD liposarcoma subtype [16–18]. Using large-scale genomic analysis, Barretina *et al.* identified YEATS4, a transcription factor also involved in p53 regulation, located on 12q15 to be frequently co-amplified with MDM2, CDK4. These three genes were shown to be functionally important in WD/DD liposarcoma [16]. Fibroblast growth factor receptor substrate 2 (FRS2), an adaptor protein that plays a critical role in FGFR signaling is also located on chromosome 12q13-15 and reported to be frequently amplified in high-grade liposarcomas [18, 19]. Genomic amplification of c-Jun and its upstream kinases is another interesting pathway that has been implicated as a mechanism of progression from WD to DD liposarcoma [20]. Snyder *et al.* showed that c-Jun protein is expressed in majority of DD liposarcomas (91%) and their accompanying WD components (59%), but only in the minority of pure WD liposarcomas (27%). When c-Jun is amplified in dedifferentiated liposarcoma, it is interspersed with amplified MDM2 on ring and giant marker chromosomes suggesting that c-Jun is amplified at a similar time in the evolution of the tumor. Our study confirms the presence of previously reported amplifications and also reveals some new recurrent and potentially actionable CNAs (*NOTCH4, DDR1, DAXX, ERBB2, BCL2, NFKB2*). Of note, c-Jun amplification was detected in one case sequenced by FM, but was not part of T200 testing.

In another study by Kanojia *et al*, liposarcoma samples (all subtypes) were analyzed using SNP arrays, whole exome sequencing and targeted exome sequencing. SNP array analyses showed amplifications in MDM2, CDK4, HMGA2, UAP1, MIR557, LAMA4, CPM, IGF2, ERBB3 and IGF1R genes [21]. Deletions were reported include deletions at chromosome 1p (RUNX3, ARID1A), chromosome 11q (ATM, CHEK1) and chromosome 13q14.2 (MIR15A, MIR16-1). Mutated genes with a false discovery rate of less than 5% were PLEC (27%), MXRA5 (21%), FAT3 (24%), NF1 (20%), MDC1 (10%), TP53 (7%) and CHEK2 (6%). It is important to point out that Kanojia *et al.* evaluated all subgroups of liposarcomas, whereas our study only analyzed WD/DD samples. Notably P53 mutations are never seen in WD/DD liposaromas.

The knowledge of the important role of MDM2 as a negative regulator of p53 has lead to preclinical and clinical studies targeting MDM2 in liposarcomas. Preclinical studies using Nutlin-3A, a selective MDM2 antagonist, stabilized p53 leading to downstream p53 dependent transcription and apoptosis in liposarcoma cells lines [22]. Ray-Coquard *et al.* published a phase I, proof of concept clinical trial of an MDM2 antagonist, RG7112 given as neoadjuvant therapy in patients with WD and DD liposarcoma [23]. Post-therapy tumor specimens demonstrated restoration of p53 and downstream p21 concentrations as well as statistically significant reduction in Ki67-positive proliferating tumor cells. Neutropenia and thrombocytopenia were the dose limiting side effects and majority experienced nausea and fatigue. During the three cycles of neoadjuvant therapy, the 70% of patients had stable disease and one patient had a partial response by RECIST. A subsequent phase Ib study of doxorubicin combined with RG7112 in soft tissue sarcomas was stopped due to increased grade 3 and 4 neutropenia and thrombocytopenia [24]. A new formulation of the MDM2 inhibitor, RG7388 was tested in a phase I study and showed p53 activation and prolonged stable disease in sarcoma patients [25]. Recently, encouraging results were seen in liposarcoma patients on a phase I study with MK-8242, a potent, orally bio-available, small-molecule inhibitor of the MDM2-p53 protein-protein interaction [26]. There are additional oral and intravenous MDM2 inhibitors being investigated in ongoing phase I studies. Eight of the patients included in our study were treated with an MDM2 inhibitor on a phase I study and experienced prolonged SD with a median time to progression of 23 months. The targeted genotyping helped confirm p53 wild-type status in these patients, which was a requirement for some of the trials. Thrombocytopenia was the major cause for treatment interruption and discontinuation. Three of these eight patients also received standard chemotherapy at some point during the course of their treatment. Regimens used included doxorubicin alone, doxorubicin with dacarbazine and cyclophosphamide, and gemcitabine and docetaxel. The median number of cycles of chemotherapy received was only two (range 2 to 6). In comparison the median number of cycles of the MDM2 inhibitor was nine (range 4 to 92) in these 3 patients. These results are promising and future studies in liposarcoma are essential to determine the right dose and combination regimens that will allow effective targeting of this pathway with acceptable toxicity.

Targeting CDK4 is also an attractive option in WD/DD liposarcoma given its frequent overexpression. Mechanistically, CDK4 phosphorylates and functionally inactivates the retinoblastoma (Rb) protein, which results in uninhibited cell cycle progression from G1 to S phase and restoring native cell cycle regulation by CDK4 inhibition could prevent uncontrolled proliferation. First generation pan-CDK inhibitors include flavopiridol and seleciclib (R-Roscovitine) that have shown preclinical efficacy especially when tumors are sequenced with cytotoxic therapy resulting in sequence-dependent cytotoxic synergy [27, 28]. Preclinical studies with the pan-CDK inhibitor flavopiridol in mouse xenograft models of soft tissue sarcoma, including DD liposarcoma showed that it could potentiate the effect of doxorubicin [29]. The phase I dose-escalation study of flavopiridol in combination with fixed dose doxorubicin showed prolonged SD in patients with WD/DD liposarcoma but given the increased hematologic toxicity, these agents have not been tested further in patients. Palbociclib (PD0332991) an oral CDK4/6 specific inhibitor was recently approved for the treatment of breast cancer in combination with tamoxifen. In the initial phase I study prolonged stable disease was also noted in four out of seven liposarcoma patients leading to a phase II study of palbociclib, specifically for patients with Rb-positive liposarcoma [30]. Of the 29 evaluable patients, 66% were progression free at 12 weeks, with a median PFS of 18 weeks. There was one partial response per RECIST. Major grade 3 and 4 toxicities were anemia (17%), neutropenia (50%) and thrombocytopenia (30%). Hematologic toxicies are common to both MDM2 and CDK4 inhibitors, thus combination trials will need to be carefully designed with close monitoring of patients for overlapping toxicities.

In this study, using the currently available clinical grade genotyping panels in WD/DD liposarcoma, 20/20 (100%) patient samples tested had *CDK4* amplified and 16/16 (100%) patient samples tested had *MDM2* amplified. Additional recurrent novel deleterious genetic changes were also identified, but further studies are needed to determine their therapeutic and pathogenetic significance. Hence, at this time the main utility of targeted sequencing in WD/DD liposarcoma patients using commercially available panels such as FM would be to identify patients for clinical trials testing agents that target the MDM2 and/or the CDK4 pathway. The sequencing results also help confirm the liposarcoma subtype based on the presence or absence of MDM2 amplification, when it is not clear on imaging and histopathology. Moving forward we hope to learn more about the genetic landscape of sarcomas through more in-depth sequencing; what additional genomic aberrations and copy number changes are important in malignant transformation and metastases and what alterations are potentially druggable. This information should then be incorporated into the available genotyping panels available for clinical use and we might perhaps have a sarcoma specific panel in the future.

## MATERIALS AND METHODS

### Patient selection

To identify WD/DD liposarcoma patients who underwent targeted next-generation sequencing a part of their treatment planning, we screened the T200/T200.1 database (in-house sequencing platform) at MDACC and the database of patients who underwent FM testing through the Sarcoma Department or the Phase I Department at MDACC from December 2012 through December 2014. Tumors that were classified as conventional atypical lipomatous tumor/WD liposarcoma, cellular atypical lipomatous tumor/ cellular WD liposarcoma and DD liposarcoma, as previously described by Evans HL were included in the analysis [31]. Mature fat with fibrous or myxoid areas and scattered atypical tumor cells with pleomorphic hyperchromatic nuclei characterize conventional atypical lipomatous tumors/WD liposarcomas. Cellular atypical lipomatous tumors/cellular WD liposarcomas are defined as an atypical lipomatous tumor, which had a non-lipogenic moderately to occasionally highly cellular fibrous or myxoid component with variable cytological atypia and a maximal mitotic rate of fewer than 5 mitotic figures/10 hpfs. Dedifferentiated liposarcoma had both conventional areas and a non-lipogenic highly to moderately cellular component with spindle or pleomorphic cells and a mitotic rate of ≥5 mitotic figures/10 hpfs. Approval for this retrospective study was obtained from the MD Anderson Institutional Review Board.

### Next-generation sequencing

Original hematoxylin and eosin slides were reviewed by an institutional pathologist to confirm the diagnosis of WD/DD liposarcoma. Archival formalin-fixed paraffin-embedded (FFPE) slides were then obtained and cut into 10 separate 5-mm sections. T200/T200.1 used hybrid capture followed by sequencing of tumor and matched non-neoplastic DNA on an Illumina HiSeq2000 platform [32]. FM sequencing, also using FFPE sections was completed in a Clinical Laboratory Improvement Amendments (CLIA)-certified laboratory using the Illumina HiSeq2000 platform (Foundation Medicine, Cambridge, MA, USA).

### T-200/T-200.1 and foundation panels

Out of the 202 genes included in the T200, the 263 genes included in the T200.1 panel and the 244 genes on the FM panel, 120 were common/overlapping targets (124 between T200 and FM, 141 between T200 and T200.1 and 171 between T200.1 and FM) (Supplementary Table 1). Variant calling for T200/T200.1: 1%–5% in most of our high coverage data (>500×), 10–15% if poorly covered (i.e., <200 × coverage). FoundationOne^®^ applies next-generation sequencing to simultaneously sequences the coding region of 315 cancer-related genes plus introns from 28 genes often rearranged or altered in cancer to a typical median depth of coverage of greater than 500×. All classes of genomic alterations, including base substitutions, insertions and deletions (indels), copy number alterations (CNAs) and rearrangements are detected.

### Fluorescence *in situ* hybridization (FISH) for MDM2

Additional molecular testing for *MDM2* amplification by FISH was performed for diagnosis confirmation on certain samples when required for diagnosis confirmation by the pathologist in a CLIA certified lab. This analysis complemented the T200 analysis since *MDM2* amplification was not part of this panel.

### Statistical analysis

Demographic data such as age at diagnosis, and sex, as well as clinical characteristics such as location of liposarcoma, histology/grade, number of surgeries and systemic therapies were collected for the sequenced patients. Descriptive statistics such as median, range and frequency distribution were used to summarize the patient information. Overall survival (OS, time from diagnosis until death) was estimated using the Kaplan–Meier method [33]. Log-rank test [34] was performed to test the difference among subgroups. A *p*-value of less than 0.05 indicated statistical significance. SAS 9.3 (SAS Institute inc, Cary, NC) was used for data analysis.

## SUPPLEMENTARY MATERIALS TABLES AND FIGURES






