# Supplementary material for: Targeted next generation sequencing of well-differentiated/dedifferentiated liposarcoma reveals novel gene amplifications and mutations
Source: Oncotarget. 2018 Apr 13;9(28):19891–9. doi: 10.18632/oncotarget.24924 (PMC5929434; doi:10.18632/oncotarget.24924)
Supplement: Supplementary file 1 [file oncotarget-09-19891-s001.pdf]

Targeted next generation sequencing of well-differentiated/ dedifferentiated liposarcoma reveals novel gene amplifications and mutations

SUPPLEMENTARY MATERIALS

Supplementary Table 1: Genes included in the panels for T200, T200.1 and Foundation Medicine. See Supplementary_Table_1

Supplementary Table 2: Gene HGNC_AAS Protein Codons Amino Position Acids. See Supplementary_Table_2

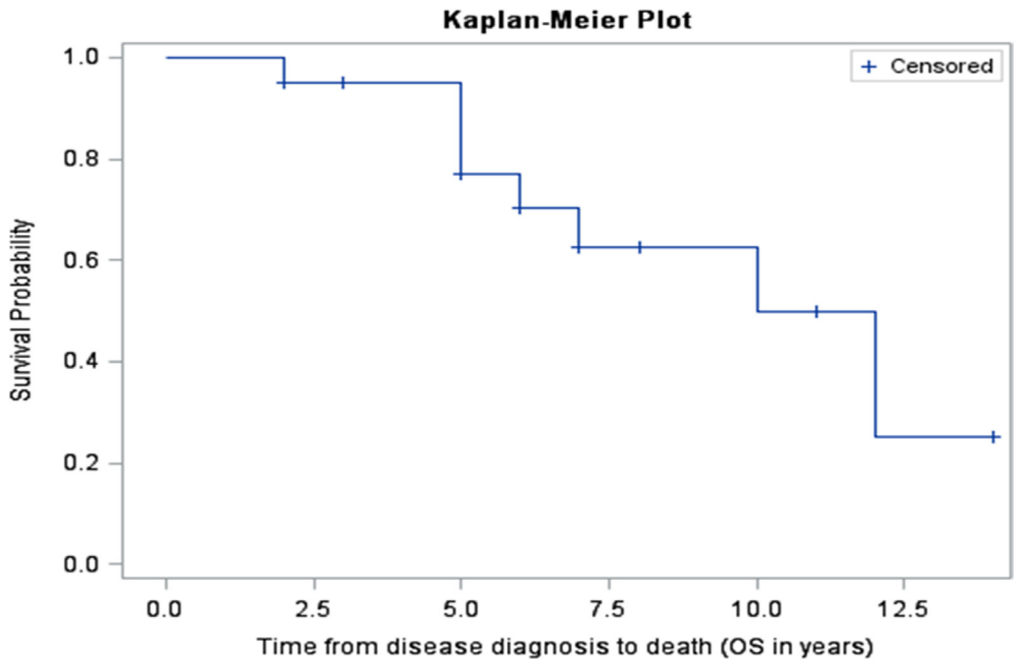

Supplementary Figure 1: Kaplan–Meier plot for estimated overall survival inall 20 patients.

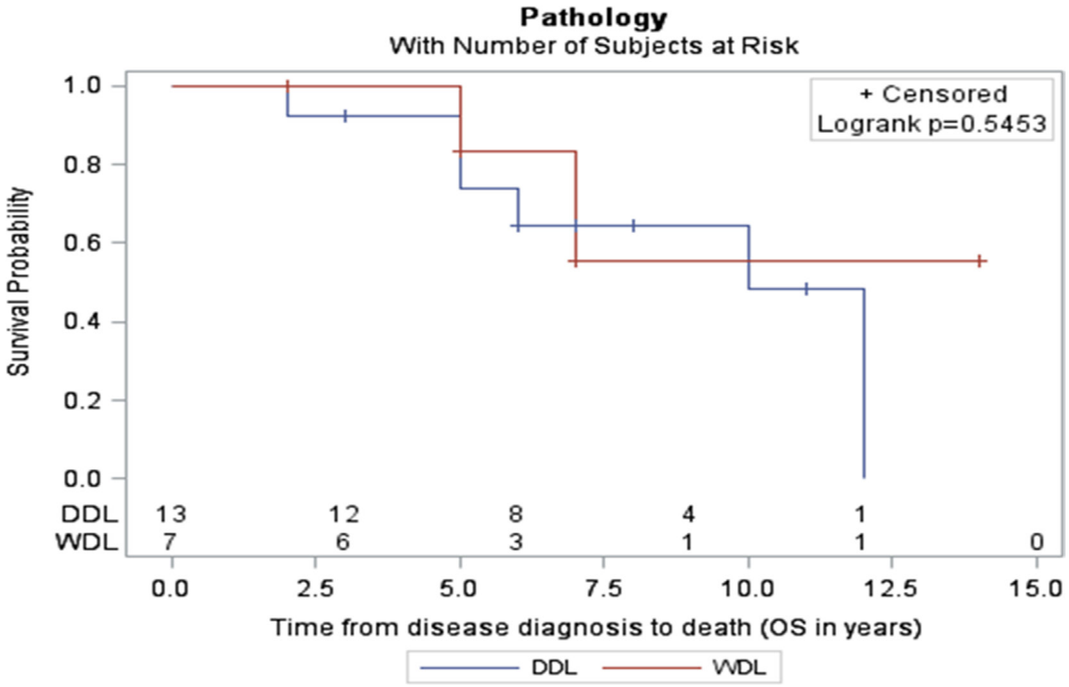

Supplementary Figure 2: Overall survival estimates based on WD vs DD histology.
