# Supplementary material for: Targeted next generation sequencing of well-differentiated/dedifferentiated liposarcoma reveals novel gene amplifications and mutations
Source: Oncotarget. 2018 Apr 13;9(28):19891–9. doi: 10.18632/oncotarget.24924 (PMC5929434; doi:10.18632/oncotarget.24924)
Supplement: Supplementary file 2 [file oncotarget-09-19891-s002.docx]

**Supplementary Table 1: Genes included in the panels for T200, T200.1 and Foundation Medicine**

| **Gene** | **Actionable** | **T200** | **T200.1** | **FoundationOne** |
| --- | --- | --- | --- | --- |
| ABL1 | Y | Y | Y | Y |
| ABL2 | Y | N | Y | N |
| ACVR1B | N | Y | Y | N |
| ACVR2A | N | N | Y | N |
| ADAMTS12 | N | Y | N | N |
| AJUBA | N | N | Y | N |
| AKAP3 | N | Y | N | N |
| AKT1 | Y | Y | Y | Y |
| AKT2 | Y | N | Y | Y |
| AKT3 | Y | N | Y | Y |
| AKTip | N | N | Y | N |
| ALK | Y | Y | Y | Y |
| APC | Y | Y | Y | Y |
| AR | Y | Y | Y | Y |
| ARAF | N | Y | Y | Y |
| ARFRP1 | N | N | N | Y |
| ARID1A | N | Y | Y | Y |
| ARID1B | N | N | Y | N |
| ARID2 | N | N | Y | Y |
| ASXL1 | N | Y | Y | Y |
| ATM | Y | Y | Y | Y |
| ATR | Y | Y | Y | Y |
| ATRX | N | Y | Y | Y |
| AURKA | Y | Y | Y | Y |
| AURKB | Y | Y | Y | Y |
| AXIN1 | N | N | Y | N |
| AXIN2 | N | N | Y | N |
| AXL | N | N | Y | Y |
| B2M | N | N | Y | N |
| B2M | N | N | Y | N |
| BAI3 | N | Y | N | N |
| BAP1 | Y | Y | Y | Y |
| BARD1 | N | N | N | Y |
| BCL11A | N | N | Y | N |
| BCL2 | Y | N | Y | Y |
| BCL2L2 | N | N | N | Y |
| BCL6 | N | N | N | Y |
| BCOR | N | N | Y | Y |
| BCORL1 | N | N | N | Y |
| BCR | Y | N | N | Y |
| BIRC2 | N | N | Y | N |
| BLM | N | N | N | Y |
| BRAF | Y | Y | Y | Y |
| BRCA1 | Y | Y | Y | Y |
| BRCA2 | Y | Y | Y | Y |
| BRIP1 | N | N | N | Y |
| BTK | Y | N | Y | Y |
| CARD11 | N | Y | Y | Y |
| CASP8 | N | Y | Y | N |
| CBFB | Y | N | N | Y |
| CBL | N | Y | Y | Y |
| CCND1 | Y | N | Y | Y |
| CCND2 | Y | N | Y | Y |
| CCND3 | Y | N | Y | Y |
| CCNE1 | Y | N | Y | Y |
| CD19 | Y | Y | N | N |
| CD79A | N | N | Y | Y |
| CD79B | N | N | Y | Y |
| CDC27 |  | N | Y | N |
| CDC73 | N | N | Y | Y |
| CDH1 | N | Y | Y | Y |
| CDH10 | N | Y | N | N |
| CDH11 | N | Y | N | N |
| CDK12 | N | N | Y | Y |
| CDK4 | Y | Y | Y | Y |
| CDK6 | Y | Y | Y | Y |
| CDK8 | N | N | N | Y |
| CDKN1B | Y | N | Y | Y |
| CDKN2A | Y | Y | Y | Y |
| CDKN2B | Y | N | N | Y |
| CDKN2C | Y | N | Y | Y |
| CEBPA | Y | Y | Y | Y |
| CHEK1 | Y | Y | Y | Y |
| CHEK2 | Y | Y | Y | Y |
| CIC | N | N | Y | Y |
| COL14A1 | N | Y | N | N |
| COL2A1 | N | N | Y | N |
| CPAMD8 | N | Y | N | N |
| CREBBP | N | Y | Y | Y |
| CRIPAK | N | Y | N | N |
| CRKL | Y | N | N | Y |
| CRLF2 | N | N | N | Y |
| CSF1R | Y | Y | Y | Y |
| CSMD1 | N | Y | N | N |
| CSMD2 | N | Y | N | N |
| CSMD3 | N | Y | N | N |
| CTCF | N | N | Y | Y |
| CTLA4 | N | N | Y | N |
| CTNNA1 | N | N | N | Y |
| CTNNB1 | Y | Y | Y | Y |
| CYLD | N | Y | Y | N |
| CYP2C19 | N | Y | Y | N |
| DAXX | N | Y | Y | Y |
| DDR1 | N | Y | N | N |
| DDR2 | Y | Y | Y | Y |
| DDX3X | N | N | Y | N |
| DICER1 | N | N | Y | N |
| DNMT3A | Y | Y | Y | Y |
| DOT1L | Y | N | N | Y |
| EGFR | Y | Y | Y | Y |
| ELF3 |  | N | Y | N |
| ELN | N | Y | N | N |
| EML4 | Y | Y | N | N |
| EMSY | N | N | N | Y |
| EP300 | N | Y | Y | Y |
| EPHA2 | N | N | Y | N |
| EPHA3 | Y | Y | Y | Y |
| EPHA5 | Y | N | Y | Y |
| EPHB1 | N | N | N | Y |
| ERBB2 | Y | Y | Y | Y |
| ERBB3 | Y | Y | Y | Y |
| ERBB4 | Y | N | Y | Y |
| ERCC2 | N | N | Y | N |
| ERCC3 | N | Y | Y | N |
| ERCC4 | N | Y | Y | N |
| ERCC5 | N | Y | Y | N |
| ERG | Y | N | N | Y |
| ESR1 | Y | Y | Y | Y |
| ETV1 | N | N | Y | Y |
| ETV4 | N | N | N | Y |
| ETV5 | N | Y | N | Y |
| ETV6 | N | N | N | Y |
| EWSR1 | N | N | N | Y |
| EZH2 | N | Y | N | Y |
| FADD | n | N | Y | N |
| FAK = PTK2 | N | N | Y | N |
| FAM123B | N | Y | Y | Y |
| FAM135B | N | Y | N | N |
| FAM46C | N | N | N | Y |
| FANCA | N | N | Y | Y |
| FANCC | N | N | N | Y |
| FANCD2 | N | N | Y | Y |
| FANCE | N | N | N | Y |
| FANCF | N | N | N | Y |
| FANCG | N | N | N | Y |
| FANCL | N | N | N | Y |
| FAT3 | N | Y | N | N |
| FBXW7 | Y | Y | Y | Y |
| FGF10 | N | N | N | Y |
| FGF14 | N | N | N | Y |
| FGF19 | N | N | N | Y |
| FGF23 | N | N | N | Y |
| FGF3 | N | N | N | Y |
| FGF4 | N | N | N | Y |
| FGF6 | N | N | N | Y |
| FGFR1 | Y | Y | Y | Y |
| FGFR2 | Y | Y | Y | Y |
| FGFR3 | Y | Y | Y | Y |
| FGFR4 | Y | Y | Y | Y |
| FH | N | N | Y | N |
| FLG | N | Y | N | N |
| FLT1 | Y | Y | Y | Y |
| FLT3 | Y | Y | Y | Y |
| FLT4 | Y | Y | Y | Y |
| FOXA1 | N | N | Y | N |
| FOXL2 | N | Y | Y | Y |
| FTO | N | N | Y | N |
| GABRA6 | N | Y | Y | N |
| GABRB3 | N | Y | N | N |
| GATA1 | N | Y | Y | Y |
| GATA2 | N | N | Y | Y |
| GATA3 | N | Y | Y | Y |
| **Gene** | **Actionable** | **T200** | **T200.1** | **FoundationOne** |
| GID4 | N | N | N | Y |
| GNA11 | Y | Y | Y | Y |
| GNA13 | N | N | N | Y |
| GNAQ | Y | Y | Y | Y |
| GNAS | Y | Y | Y | Y |
| GPR124 | N | N | N | Y |
| GRIN2A | N | N | N | Y |
| GSK3B | N | N | Y | N |
| GSK3B | Y | N | N | Y |
| H3F3A | N | N | Y | N |
| HDAC9 | Y | Y | N | N |
| HEATR7B2 | N | Y | N | N |
| HGF | N | Y | N | Y |
| HIST1H3B | N | N | Y | N |
| HLA-A | N | N | Y | N |
| HMCN1 | N | Y | N | N |
| HNF1A | N | Y | Y | N |
| HNF1B | N | Y | N | N |
| HRAS | Y | Y | Y | Y |
| HSP90AB1 | Y | N | Y | N |
| HYDIN | N | Y | N | N |
| IDH1 | N | Y | Y | Y |
| IDH2 | N | Y | Y | Y |
| IGF1R | Y | Y | Y | Y |
| IKBKE | N | N | N | Y |
| IKZF1 | N | Y | N | Y |
| IL6R | N | Y | N | N |
| IL7R | Y | N | Y | Y |
| INHBA | N | N | N | Y |
| IRF4 | N | N | N | Y |
| IRS1 | N | Y | N | N |
| IRS2 | N | N | N | Y |
| ITGA4 | N | Y | N | N |
| JAK1 | Y | Y | Y | Y |
| JAK2 | Y | Y | Y | Y |
| JAK3 | Y | Y | Y | Y |
| JUN | N | N | N | Y |
| KAT6A | N | N | N | Y |
| KCNB2 | N | Y | N | N |
| KDM5A | N | N | N | Y |
| KDM5C | N | N | Y | Y |
| KDM6A | N | Y | Y | Y |
| KDR | Y | Y | Y | Y |
| KEAP1 | N | N | Y | Y |
| KIT | Y | Y | Y | Y |
| KLHL6 | N | N | N | Y |
| KRAS | Y | Y | Y | Y |
| LAMA1 | N | Y | N | N |
| LPHN3 | N | Y | N | N |
| LRP1 | N | Y | N | N |
| LRP1B | N | Y | Y | Y |
| LRP2 | N | Y | N | N |
| MAP2K1 | Y | Y | Y | Y |
| MAP2K2 | Y | N | Y | Y |
| MAP2K4 | Y | Y | Y | Y |
| MAP3K1 | Y | Y | Y | Y |
| MAP3K13 | N | N | Y | N |
| MAP3K4 | Y | Y | Y | N |
| MAPK1 | Y | N | Y | N |
| MCL1 | N | N | Y | Y |
| MDM2 | Y | N | Y | Y |
| MDM4 | Y | N | N | Y |
| MDN1 | N | Y | N | N |
| MECOM | N | Y | N | N |
| MED12 | N | N | Y | Y |
| MEF2B | N | N | N | Y |
| MEN1 | Y | Y | Y | Y |
| MET | Y | Y | Y | Y |
| MITF | N | Y | Y | Y |
| MLH1 | N | Y | Y | Y |
| MLL | Y | N | Y | Y |
| MLL2 | Y | Y | Y | Y |
| MLL3 | Y | Y | Y | N |
| MPL | Y | Y | Y | Y |
| MRE11A | N | N | N | Y |
| MSH2 | N | Y | Y | Y |
| MSH6 | N | Y | Y | Y |
| MST1 | N | N | Y | N |
| MST1R | N | N | Y | N |
| MTOR | Y | Y | Y | Y |
| MUTYH | N | N | Y | Y |
| MYC | Y | N | Y | Y |
| MYCL1 | N | N | N | Y |
| MYCN | N | N | N | Y |
| MYD88 | N | Y | Y | Y |
| NAV3 | N | Y | N | N |
| NBN | N | N | Y | N |
| NCOR1 | N | Y | Y | N |
| NF1 | Y | Y | Y | Y |
| NF2 | Y | Y | Y | Y |
| NFE2L2 | N | N | Y | Y |
| NFKB2 | N | Y | N | N |
| NFKBIA | N | N | N | Y |
| NKX2-1 | N | N | Y | Y |
| NOTCH1 | Y | Y | Y | Y |
| NOTCH2 | Y | Y | Y | Y |
| NOTCH3 | Y | Y | Y | N |
| NOTCH4 | Y | Y | Y | N |
| NPM1 | Y | Y | Y | Y |
| NRAS | Y | Y | Y | Y |
| NSD1 | N | Y | Y | N |
| NTRK1 | Y | N | Y | Y |
| NTRK2 | Y | N | N | Y |
| NTRK3 | Y | N | Y | Y |
| NUP93 | N | N | N | Y |
| PAK3 | N | N | N | Y |
| PALB2 | N | Y | Y | Y |
| PAPPA2 | N | Y | N | N |
| PAX5 | N | Y | Y | Y |
| PBRM1 | N | Y | Y | Y |
| PCDH15 | N | Y | N | N |
| PCLO | N | Y | N | N |
| PDCD1 | N | N | Y | N |
| PDGFRA | Y | Y | Y | Y |
| PDGFRB | Y | Y | Y | Y |
| PDK1 | Y | N | N | Y |
| pdl1 | N | N | Y | N |
| PHF6 | N | N | Y | N |
| PIK3CA | Y | Y | Y | Y |
| PIK3CG | Y | Y | Y | Y |
| PIK3R1 | Y | Y | Y | Y |
| PIK3R2 | Y | N | N | Y |
| PIKFYVE | N | Y | N | N |
| PKHD1 | N | Y | N | N |
| PKHD1L1 | N | Y | N | N |
| PLCG1 | N | N | Y | N |
| PMS2 | N | N | Y | N |
| POLE | N | N | Y | N |
| PPM1D | N | N | Y | N |
| PPP1R3A | N | Y | Y | N |
| PPP2R1A | N | Y | Y | Y |
| PPP2R4 | N | Y | N | N |
| PRDM1 | N | Y | Y | Y |
| PREX2 | N | N | Y | N |
| PRG4 | N | N | Y | N |
| PRKAR1A | N | N | N | Y |
| PRKDC | N | N | N | Y |
| PRSS1 | N | Y | N | N |
| PTCH1 | Y | Y | Y | Y |
| PTEN | Y | Y | Y | Y |
| PTK2 | Y | Y | N | N |
| PTPN11 | Y | Y | Y | Y |
| PTPRB | N | N | Y | N |
| RAC1 | N | N | Y | N |
| RAD50 | N | N | N | Y |
| RAD51 | Y | Y | Y | Y |
| RAD51C | N | N | Y | N |
| RAF1 | Y | Y | Y | Y |
| RARA | Y | N | Y | Y |
| RB1 | N | Y | Y | Y |
| RELN | N | Y | N | N |
| RET | Y | Y | Y | Y |
| RHOH | N | N | Y | N |
| RICTOR | Y | N | Y | Y |
| RIMS2 | N | Y | N | N |
| RNF213 | N | Y | N | N |
| RNF43 | N | N | Y | Y |
| ROS1 | Y | N | Y | Y |
| RPTOR | N | N | Y | Y |
| RUNX1 | Y | Y | Y | Y |
| RUNX1T1 | N | Y | Y | N |
| RYR2 | N | Y | N | N |
| SDHB | N | N | Y | N |
| SDHC | N | N | Y | N |
| SDHD | N | N | Y | N |
| SETBP1 | N | N | Y | N |
| SETD2 | N | Y | Y | Y |
| SF3B1 | N | N | Y | Y |
| SMAD2 | N | N | Y | Y |
| SMAD3 | N | N | Y | N |
| SMAD4 | N | Y | Y | Y |
| SMARCA2 | N | N | Y | N |
| SMARCA4 | Y | Y | Y | Y |
| SMARCB1 | Y | Y | Y | y |
| SMARCD1 | N | N | Y | N |
| SMC1A | N | N | Y | N |
| SMC3 | N | N | Y | N |
| SMO | Y | Y | Y | Y |
| SOCS1 | N | N | Y | Y |
| SOS1 | N | Y | Y | N |
| SOX10 | N | N | N | Y |
| SOX2 | N | N | N | Y |
| SOX9 | N | N | Y | N |
| SPEN | N | Y | Y | Y |
| SPOP | N | Y | Y | Y |
| SPTA1 | N | Y | N | N |
| SRC | Y | N | Y | Y |
| SRSF2 | N | N | Y | N |
| STAG2 | N | N | Y | Y |
| STAT3 | N | N | Y | N |
| STAT4 | N | N | N | Y |
| STK11 | Y | Y | Y | Y |
| STK19 | N | N | Y | N |
| SUFU | N | N | Y | Y |
| SYK | Y | Y | Y | N |
| SYNE1 | N | Y | N | N |
| SYNE2 | N | Y | N | N |
| TBC1D4 | N | Y | N | N |
| TBC1D4 | N | N | Y | N |
| TBX3 | N | N | Y | N |
| TERT | N | N | Y | N |
| TET2 | Y | Y | Y | Y |
| TGFb1 | N | Y | Y | N |
| TGFBR1 | N | N | Y | N |
| TGFBR2 | N | Y | Y | Y |
| TMPRSS2 | N | N | N | Y |
| TNF | N | N | Y | N |
| TNFAIP3 | N | Y | Y | Y |
| TNFRSF14 | N | N | N | Y |
| TOP1 | Y | Y | Y | Y |
| TOP2A | N | Y | Y | N |
| TP53 | N | Y | Y | Y |
| TSC1 | Y | Y | Y | Y |
| TSC2 | Y | Y | Y | Y |
| TSHR | N | Y | Y | Y |
| U2AF1 | N | N | Y | N |
| USH2A | N | Y | N | N |
| VEGFA | N | N | Y | N |
| VHL | Y | Y | Y | Y |
| VHL | N | N | Y | N |
| WHSC1 | N | Y | N | N |
| WHSC1L1 | N | N | Y | N |
| WISP3 | N | N | N | Y |
| WT1 | Y | Y | Y | Y |
| XPO1 | Y | N | Y | Y |
| XPO1 | N | N | Y | N |
| ZNF217 | N | N | N | Y |
| ZNF238 | N | Y | N | N |
| ZNF536 | N | Y | N | N |
| ZNF703 | N | N | N | Y |
| ZRSR2 | N | N | Y | N |
