# Supplementary material for: Targeted next generation sequencing of well-differentiated/dedifferentiated liposarcoma reveals novel gene amplifications and mutations
Source: Oncotarget. 2018 Apr 13;9(28):19891–9. doi: 10.18632/oncotarget.24924 (PMC5929434; doi:10.18632/oncotarget.24924)
Supplement: Supplementary file 3 [file oncotarget-09-19891-s003.docx]

**Patient** **1**

**T200** **Mutations**

**Freq**

**Gene** **HGNC_AAS** **Protein** **Codons** **Amino** **Position** **Acids** **Exon** **Allele** **Confidence** **Coverage** **Nucleotide** **Position** **dbSNP** **Condel** **Uploaded** **Variation** **P**

CTNNB1 CTNNB1_R151H 151 cGt/cAt R/H 4/15 5.33% low 75 452 NA deleterious(0.635) 3_41266655_G/A p

FAT3 FAT3_T2440M 2440 aCg/aTg T/M 9/27 5.63% low 71 7319 NA neutral(0.030) 11_92533498_C/T b

GNAS - 547 cgC/cgT R 1/2 8.89% low 45 1641 NA - 20_57429961_C/T -

MECOM MECOM_R208C 208 Cgc/Tgc R/C 7/15 26.09% medium 69 622 NA deleterious(0.902) 3_168834474_C/T p

TBC1D4 TBC1D4_V41I 41 Gtt/Att V/I 1/21 7.27% low 55 121 NA neutral(0.034) 13_76055783_G/A b

ZNF536 ZNF536_T688M 688 aCg/aTg T/M 2/5 5.33% low 75 2063 NA deleterious(0.911) 19_30936532_C/T p

**T200** **CNA**

**Gene** **Copy** **#** **Alteration** **Estimate** **Copy** **#** **SkipReporting?** **CGMB** **Sent?** **Gene** **Copy** **#** **Alteration** **CN** **SkipReporting?**

ABL1 **H.AMP,AMP** 4.8,2.7 N Y AURKB **H.AMP** 4.8 N

DDR1 **H.AMP** 4.2 N Y ERBB2 **H.AMP,AMP** 4.2,2.8 N

FLT3 **H.DEL** 0.8 N Y FLT4 **NORM,H.AMP** 2.2,4 N

ITGA4 **H.AMP,NORM** 4.4,2.3 N Y LRP2 **NORM,H.AMP** 1.7,4.4 N

NAV3 **H.AMP,NORM** 4.9,1.8 N Y NFKB2 **H.AMP** 4.5 N

PPP2R4 **H.AMP** 4.8 N Y PTCH1 **AMP,H.AMP** 2.7,4.8 N

ACVR1B AMP 3.1 N Y AKT1 AMP 3.5 N

ASXL1 AMP 2.5 N Y ATM NORM,DEL 21.5 N

BRCA2 DEL 1.1 N Y CARD11 AMP,NORM 3.2,2 N

CDH11 DEL 1.2 N Y CDK6 AMP 2.8 N

CPAMD8 AMP,NORM 3.4,2.3,3.1 N Y CREBBP AMP,NORM 3.6,2.2,3.3 N

CSMD1 DEL 1.2 N Y CSMD2 AMP,NORM 2.9,1.7 N

CYLD DEL 1.2 N Y CYP2C19 DEL 1.2 N

ERBB3 AMP,NORM 3.1,2.2 N Y ERCC3 AMP 2.9 N

FAM135B DEL 1.2 N Y FAT3 DEL,NORM 1.4,2 N

FGFR2 DEL 1.4 N Y FGFR3 AMP 3.1 N

GNA11 AMP 3.4 N Y GNAQ AMP 2.7 N

HNF1A NORM,AMP 1.8,3.4 N Y IDH2 AMP 3.5 N

JAK2 DEL 1 N Y JAK3 AMP 3.1 N

KIT DEL 1.5 N Y LPHN3 DEL 1.5 N

MDN1 DEL,AMP 1.4,2.8 N Y MITF NORM,DEL 2.2,1 N

MTOR NORM,AMP 22.9 N Y MYD88 DEL 1.4 N

PAX5 AMP 2.7 N Y PBRM1 DEL,NORM 1.5,2.2 N

PDGFRA DEL 1.5 N Y PDGFRB AMP 3.1 N

PPP2R1A AMP 3.1 N Y PRDM1 AMP 2.8 N

RB1 DEL 1.1 N Y RELN DEL,NORM 1.2,1.6 N

RNF213 NORM,AMP 2.1,2.7 N Y RUNX1 AMP 3.1 N

SMARCA4 AMP 3.4 N Y SMO AMP 2.8 N

SYNE1 AMP,NORM 2.8,2,1.6 N Y SYNE2 DEL,NORM 1.4,2.2 N

TGFB1 AMP 3.1 N Y TNFAIP3 AMP 2.8 N

TSC1 AMP 2.7 N Y TSC2 AMP 3.6 N

WHSC1 AMP,NORM 3.1,1.6,2.5 N Y ZNF536 AMP 3.1 N

**Patient** **2**

**T200** **Mutations**

**Freq**

**Gene** **HGNC_AAS** **Protein** **Codons** **Amino** **Position** **Acids** **Exon** **Allele** **Confidence** **Coverage** **Nucleotide** **Position** **dbSNP** **Condel** **Uploaded** **Variation** **P**

HLA-C HLA-C_T224P 224 Acg/Ccg T/P 3/8 19.72% low 71 670 rs1050685 - 6_31238910_T/G b

HLA-C HLA-C_T224M 224 aCg/aTg T/M 3/8 19.44% low 72 671 rs1050686 - 6_31238909_G/A b

PMS2 PMS2_E398A 398 gAa/gCa E/A 11/15 29.23% medium 414 1193 NA neutral(0.060) 7_6027203_A/C b

**T200** **CNA**

**Gene** **Copy** **#** **Alteration** **Estimate** **Copy** **#** **SkipReporting?** **CGMB** **Sent?** **Gene** **Copy** **#** **Alteration** **CN** **SkipReporting?**

ATP5EP2 **H.DEL** 0.8 N Y ATRX **NORM,AMP,H.AMP** 2,3.9,5.6,2.2 N

BTK **NORM,H.DEL** 2.2,0.9 N Y CBWD1 **H.DEL,NORM,DEL** 0.9,1.8,1.2 N

CDX2 **H.DEL** 0.8 N Y CEBPA **H.DEL** 1 N

F8 **H.AMP** 5.6 N Y F8A1 **H.AMP** 5.6 N

FLT4 **H.DEL** 1 N Y FOXD4 **H.DEL** 0.9 N

GAB3 **H.AMP** 5.6 N Y GPR143 **H.DEL** 1 N

HELT **H.DEL** 0.8 N Y IFNA1 **H.DEL** 0.9 N

IFNA5 **H.DEL** 0.9 N Y IFNA6 **H.DEL** 0.9 N

IQSEC3 **H.DEL** 0.9 N Y IRAK1 **H.DEL** 0.9 N

LAGE3 **H.DEL** 0.9 N Y LYZ **H.AMP** 4.2 N

NEFM **H.DEL** 0.9 N Y NINJ2 **H.DEL,DEL** 0.9,1.1 N

PDX1 **H.DEL** 0.8 N Y PPP2R2A **H.DEL,DEL** 0.9,1.5 N

RAD51 **H.DEL** 0.9 N Y ROS1 **H.AMP** 13.2 N

SLC6A13 **H.DEL** 0.9 N Y SMO **DEL,H.DEL** 1.2,0.9 N

STC1 **H.DEL,DEL** 0.9,1.2 N Y TBL1X **H.DEL** 1 N

TSPAN31 **H.AMP** 49.8 N Y WASH1 **H.DEL,NORM,DEL,AMP** 0.9,1.8,1.2,0.9,2.2,1.4,2.7 N

ZRSR2 **H.DEL,NORM,DEL** 1,1.6,1.1 N Y ABL1 AMP,NORM 2.7,1.7 N

ADIPOR2 DEL 1.1 N Y AIFM3 DEL 1.3 N

AKT3 AMP,NORM 2.6,1.7 N Y AMER1 DEL 1.4 N

AR DEL 1.4 N Y ASPSCR1 AMP,NORM 3.1,1.7 N

AXIN1 DEL 1.3 N Y AXL DEL 1.5 N

BRAF DEL 1.2 N Y BRCA2 NORM,DEL 1.7,1.2 N

C19ORF12 DEL 1.4 N Y C1ORF150 AMP 2.8 N

C4ORF47 DEL 1.3 N Y C9ORF131 DEL 1.4 N

CABLES2 DEL 1.1 N Y CACNA1A DEL 1.4 N

CCDC66 NORM,AMP 1.9,2.6,2 N Y CCND2 DEL 1.1 N

CD79A DEL 1.5 N Y CDC42EP4 AMP 3.3 N

CHEK1 DEL 1.2 N Y CHL1 DEL 1.1 N

CLPTM1L DEL 1.5 N Y CNTN4 DEL 1.1 N

CREBBP DEL,NORM 1.3,1.8 N Y CTNNB1 DEL 1.1 N

DMRTA1 DEL 1.4 N Y DNAJB5 DEL 1.4 N

DOK6 AMP 2.8 N Y ERBB2 NORM,DEL 1.7,1.4 N

EZH2 DEL 1.2 N Y FADD DEL 1.3 N

FBRS NORM,DEL 1.8,1.4 N Y FGFR3 DEL 1.2 N

FLT1 DEL 1.1 N Y FOXL2 DEL 1.5 N

GAGE2A DEL 1 N Y GATA2 DEL 1.5 N

GNAQ AMP 2.7 N Y GNAS DEL,NORM 1.3,2.3 N

GPR35 DEL 1.2 N Y GSC2 DEL 1.3 N

HNF1A DEL,NORM 1.3,2 N Y HRAS DEL 1 N

RF2 DEL 1.4 N Y IRS4 DEL 1.5 N

KANK1 DEL 1.2 N Y KATNAL1 DEL 1.1 N

KDM6A NORM,AMP 2.2,4 N Y KEAP1 DEL 1.4 N

KIAA1430 DEL 1.3 N Y KIF1A DEL,NORM 1.2,1.5 N

LRP2BP DEL 1.3 N Y MAP2K2 DEL 1.4 N

MET DEL 1.2 N Y MLL DEL 1.1 N

**Gene** **Copy** **#** **Alteration** **Estimate** **Copy** **#** **SkipReporting?** **CGMB** **Sent?** **Gene** **Copy** **#** **Alteration** **CN** **SkipReporting?**

MST1R DEL 1.4 N Y MTAP NORM,DEL 2.2,1.4 N

NETO1 AMP,NORM 2.8,1.7 N Y NF1 AMP 2.6 N

NOTCH1 DEL 1.2 N Y NOTCH3 DEL 1.4 N

ﬁle:///Users/nsomaiah/Desktop/liposarcoma/T200_FO_hong_manuscript/IPCT%20T200%20Results%20Full_Internal.html Page 1 of 4

NSD1 DEL,NORM 1.4,2,1.7 N Y ORAOV1 NORM,DEL 1.8,1.3 N

PAX9 DEL,NORM 1.3,1.9 N Y PIK3CG DEL 1.2 N

POLE DEL 1.4 N Y POU5F1B DEL 1.2 N

PPP1R3A DEL 1.2 N Y PTCH1 AMP 2.7 N

PTPRG NORM,DEL 2,1.1,2.2 N Y RAD51C AMP 3.8 N

RFX3 DEL 1.2 N Y RNF43 AMP 2.7,3.8 N

SEPT5 DEL 1.3 N Y SETBP1 AMP 3.2 N

SLC6A18 DEL 1.1 N Y SLC6A19 DEL 1.1 N

SMARCA2 DEL 1.2 N Y SMARCA4 DEL 1.4 N

SNTN DEL 1 N Y SNX25 DEL 1.3 N

STK11 DEL 1.4 N Y SYK AMP 2.7 N

TERT DEL 1.1 N Y TGFB1 DEL 1.5 N

TOP1 AMP,NORM 2.8,1.9 N Y TSC2 DEL 1.3 N

UFSP2 DEL 1.3 N Y USP12 DEL 1.4 N

WNK1 DEL 1.1 N Y

**Patient** **3**

**T200** **Mutations**

**Freq**

**Gene** **HGNC_AAS** **Protein** **Codons** **Amino** **Position** **Acids** **Exon** **Allele** **Confidence** **Coverage** **Nucleotide** **Position** **dbSNP** **Condel** **Uploaded** **Variation** **P**

EML4 EML4_W729L 729 tGg/tTg W/L 20/23 35.38% medium 1173 2186 NA deleterious(0.935) 2_42552638_G/T p

EML4_W729L

EML4 729 tGg/tTg W/L 20/23 35.38% medium 1173 2186 NA deleterious(0.935) 2_42552638_G/T p

**T200** **CNA**

**Gene** **Copy** **#** **Alteration** **Estimate** **Copy** **#** **SkipReporting?** **CGMB** **Sent?** **Gene** **Copy** **#** **Alteration** **CN** **SkipReporting?**

CDK4 **H.AMP** 38.2 N Y MDN1 **NORM,H.AMP** 1.9,4 N

PTPN11 **H.AMP,NORM** 4.4,1.8 N Y CRIPAK DEL 1.5 N

FLT4 NORM,DEL 1.9,1.5 N Y GNAS NORM,AMP 22.9 N

NOTCH2 NORM,AMP 2.1,2.7,1.7 N Y PKHD1 AMP,NORM 31.9 N

TGFBR2 AMP 2.8 N Y TNFAIP3 AMP 2.8 N

**Gene** **Copy** **#** **Alteration** **Estimate** **Copy** **#** **SkipReporting?** **CGMB** **Sent?** **Gene** **Copy** **#** **Alteration** **CN** **SkipReporting?**

ARAF **H.AMP,AMP** 4.9,3.7 N Y ARAF **H.AMP,AMP** 4.9,3.7 N

FLT4 **NORM,H.DEL** 1.6,0.9 N Y FLT4 **NORM,H.DEL** 1.6,0.9 N

KDM6A **H.AMP** 4.9 N Y KDM6A **H.AMP** 4.9 N

AKT1 DEL 1.4 N Y AKT1 DEL 1.4 N

CARD11 DEL,NORM 1.3,1.8 N Y CARD11 DEL,NORM 1.3,1.8 N

CRIPAK DEL 1.1 N Y CRIPAK DEL 1.1 N

ELN NORM,DEL 1.8,1.4,1.8 N Y ELN NORM,DEL 1.8,1.4,1.8 N

FLG DEL 1.3 N Y FLG DEL 1.3 N

GNAQ AMP 2.9 N Y GNAQ AMP 2.9 N

HNF1A DEL 1.1 N Y HNF1A DEL 1.1 N

JAK3 DEL 1.1 N Y JAK3 DEL 1.1 N

NFKB2 NORM,DEL 1.7,1.2 N Y NFKB2 NORM,DEL 1.7,1.2 N

NOTCH2 NORM,DEL 1.7,2.4,1.3 N Y NOTCH2 NORM,DEL 1.7,2.4,1.3 N

NOTCH4 NORM,DEL 1.9,1.4 N Y NOTCH4 NORM,DEL 1.9,1.4 N

RET NORM,DEL 1.5,1.1,1.7 N Y RET NORM,DEL 1.5,1.1,1.7 N

STK11 DEL 1.5 N Y STK11 DEL 1.5 N

**CLIA** **Result**

**Gene** **Result** **Codons** **Amino** **Acid** **Exon** **SAccession** **MAccession** **Comments** **Gene** **Result** **Codons**

**CMS46**

KDR Variant Poss. Germline Polymorphism 482 C/R S-13-007791 M-13-001801

KDR Variant Poss. Germline Polymorphism 482

**Patient** **4**

**T200** **Mutations**

**Freq**

**Gene** **HGNC_AAS** **Protein** **Codons** **Amino** **Position** **Acids** **Exon** **Allele** **Confidence** **Coverage** **Nucleotide** **Position** **dbSNP** **Condel** **Uploaded** **Variation** **P**

BRCA2 BRCA2_R3381K 3381 aGa/aAa R/K 27/28 5.97% low 67 10142 NA neutral(0.001) 13_32972792_G/A

CRIPAK CRIPAK_H132D 132 Cat/Gat H/D 1/1 5.83% low 120 394 rs78906219 - 4_1388693_C/G

CSMD3 CSMD3_P627S 627 Cca/Tca P/S 13/71 5.56% low 108 1879 NA deleterious(0.811) 8_113812484_C/T

EPHA3 EPHA3_W924R 924 Tgg/Cgg W/R 16/17 6.47% low 139 2770 rs35124509 neutral(0.037) 3_89521693_T/C

KDM6A KDM6A_Q160K 160 Caa/Aaa Q/K 15/30 4.3% low 93 478 NA deleterious(0.568) X_44920626_C/A

LRP2 LRP2_E3848K 3848 Gaa/Aaa E/K 61/79 3.74% low 107 11542 NA deleterious(0.700) 2_170025142_G/A

NF1 NF1_C845F 845 tGt/tTt C/F 21/58 4.26% low 94 2534 NA neutral(0.456) 17_29556167_G/T

NF2 NF2_K20* 20 Aag/Tag K/* 1/16 52.53% high 396 58 NA - 22_30000045_A/T

PBRM1 PBRM1_N639K 639 aaC/aaA N/K 15/28 5.48% low 73 1917 NA deleterious(0.746) 3_52643883_C/A

PBRM1 PBRM1_V1150I 1150 Gta/Ata V/I 21/28 6.15% low 65 3448 NA neutral(0.000) 3_52610704_G/A

PPP1R3A PPP1R3A_C788Y 788 tGt/tAt C/Y 4/4 7.84% low 51 2363 NA deleterious(0.935) 7_113518784_G/A

SETD2 SETD2_T2038I 2038 aCt/aTt T/I 15/22 3.9% low 154 6113 NA neutral(0.319) 3_47103833_C/T

TOP2A TOP2A_C1088Y 1088 tGt/tAt C/Y 30/41 5.56% low 72 3263 NA neutral(0.007) 17_38556297_G/A

**T200** **CNA**

**Gene** **Copy** **#** **Alteration** **Estimate** **Copy** **#** **SkipReporting?** **CGMB** **Sent?** **Gene** **Copy** **#** **Alteration** **CN** **SkipReporting?** **CGMB** **Sent?** **Gene** **Copy** **#** **Alteration** **Estimate** **Copy** **#** **SkipReporting?** **CGMB** **Sen**

AKAP3 **H.DEL** 0.8 N Y AKT1 **H.AMP** 7.9 N Y

AURKB **H.AMP** 5 N Y BAP1 **H.AMP,AMP** 12.1,3,9.1 N Y

CARD11 **H.AMP,NORM** 5.7,2.1 N Y CASP8 **H.DEL** 1 N Y

CDH10 **H.DEL** 0.7 N Y CDH11 **DEL,H.AMP** 1.1,11.9 N Y

COL14A1 **H.DEL,NORM** 0.8,1.6 N Y CPAMD8 **H.AMP** 9.7,5.6 N Y

CSF1R **H.AMP** 4.3 N Y CSMD1 **DEL,H.AMP** 1.2,8.2 N Y

DAXX **H.AMP** 5.5 N Y DDR1 **H.AMP** 5.5 N Y

ERBB2 **H.AMP** 4.8 N Y ERCC5 **H.AMP,H.DEL** 5.1,0.8 N Y

FGFR3 **H.AMP** 7.2 N Y FGFR4 **H.AMP** 8.1 N Y

FLT4 **H.AMP** 7.2 N Y GATA1 **H.AMP** 5.3 N Y

HNF1A **H.AMP** 5.4 N Y HNF1B **H.AMP** 4.8 N Y

IGF1R **H.AMP,NORM** 6.5,2.1 N Y IRS1 **H.DEL** 1 N Y

JAK3 **H.AMP** 5.6 N Y KRAS **H.DEL** 0.8 N Y

MAP2K4 **H.AMP,DEL** 5,1.2 N Y MEN1 **H.AMP** 6.9 N Y

NF1 **DEL,H.AMP** 1.2,4.8 N Y NFKB2 **H.AMP** 4.1,9.7 N Y

NOTCH4 **H.AMP** 5.5 N Y NSD1 **DEL,H.AMP** 1.4,7.2 N Y

PCDH15 **H.DEL** 0.9 N Y PDGFRB **H.AMP** 4.3 N Y

PPP2R1A**H.AMP** 5.6 N Y PRSS1 **H.AMP,DEL** 4.6,1.2 N Y

RB1 **H.DEL** 0.7 N Y RET **H.AMP,H.DEL** 5.4,0.9 N Y

SMARCA4 **H.AMP** 10.3,4.9,9.7 N Y SMO **H.AMP** 6 N Y

TGFB1 **H.AMP** 5.6 N Y TP53 **H.AMP** 5 N Y

ZNF536 **H.AMP** 5.6 N Y ABL1 AMP 3.2 N Y

ASXL1 AMP 3.4 N Y ATM DEL 1.1 N Y

AURKADEL 1.1 N Y BAI3 DEL 1 N Y

CDK6 DEL 1.1 N Y CDKN2AAMP 3.2 N Y

CYLD DEL 1.1 N Y DDR2 DEL 1.4 N Y

EPHA3 DEL 1 N Y ERBB3 DEL,AMP 1.4,2.8 N Y

EZH2 DEL 1.2 N Y FAT3 DEL 1.1 N Y

GABRA6 DEL 1.1 N Y GNAQ AMP 3.2 N Y

ﬁle:///Users/nsomaiah/Desktop/liposarcoma/T200_FO_hong_manuscript/IPCT%20T200%20Results%20Full_Internal.html Page 2 of 4

MCN1 DEL 1.4,1 N Y KCNB2 AMP 2.6 N Y

IT DEL 1.1 N Y LAMA1 DEL,AMP 1.5,3.5,1.4 N Y

AP3K1 NORM,DEL 2,1 N Y MAP3K4 DEL 1.1 N Y

ET DEL 1.1 N Y MLL2 AMP 2.5 N Y

**Gene** **Copy** **#** **Alteration** **Estimate** **Copy** **#** **SkipReporting?** **CGMB** **Sent?** **Gene** **Copy** **#** **Alteration** **CN** **SkipReporting?**

MSH6 DEL 1.1 N Y NCOR1 DEL 1.2 N

PAPPA2 DEL 1.4 N Y PAX5 AMP 3.2 N

PIK3CA DEL 1 N Y PIK3CG DEL 1.1 N

PPP1R3A DEL 1.1 N Y PPP2R4 AMP 3.2 N

RELN DEL 1.1 N Y RNF213 AMP 3 N

RYR2 DEL 1 N Y SMAD4 DEL 1.4 N

SPOP DEL 1.3 N Y SPTA1 DEL 1.4 N

SYNE2 DEL,AMP 1.1,2.9 N Y TET2 DEL 1.1 N

TSC1 AMP 3.2 N Y TSHR AMP,DEL 2.9,1 N

ZNF238 DEL 1 N Y

**CLIA** **Result**

**Gene** **Result** **Codons** **Amino** **Acid** **Exon** **SAccession** **MAccession** **Comments** **Gene** **Result** **Codons**

**CMS46**

KDR Variant Prob. Germline 482 C/R S-12-057271 M-13-005683 Polymorphism

**BioMarkers**

**Patient** **5**

**T200** **Mutations**

**Freq**

**Gene** **HGNC_AAS** **Protein** **Codons** **Amino** **Position** **Acids** **Exon** **Allele** **Confidence** **Coverage** **Nucleotide** **Position** **dbSNP** **Condel** **Uploaded** **Variation** **P**

CPAMD8 CPAMD8_G1879R 1879 Ggg/Agg G/R 42/42 37.9% medium 1169 5635 NA neutral(0.296) 19_17004116_G/A b

**T200** **CNA**

**Gene** **Copy** **#** **Alteration** **Estimate** **Copy** **#** **SkipReporting?** **CGMB** **Sent?** **Gene** **Copy** **#** **Alteration** **CN** **SkipReporting?**

AKT1 **H.AMP** 5.2 N Y ARAF **H.AMP** 5.1 N

GATA1 **H.AMP** 5.1 N Y HMCN1 **H.AMP,NORM,AMP** 5.8,1.8,3.1,1.8,3.8,1.8 N

LRP1 **H.AMP,AMP** 20.3,3 N Y NOTCH1 **H.AMP** 4.1 N

ADAMTS12 AMP,DEL 2.5,1.4 N Y APC DEL 1.4 N

ATM DEL 1.5 N Y ATRX DEL 1.5 N

BAP1 AMP 2.9 N Y BRAF DEL,AMP 1.3,2.6 N

CBL DEL 1.5 N Y CD19 AMP 2.7 N

CDH11 NORM,AMP 22.8 N Y CDK6 DEL 1.4 N

CHEK2 AMP,NORM 2.8,1.7,2.4 N Y COL14A1 DEL 1.1 N

CRIPAK AMP 2.7 N Y CSF1R AMP 2.8 N

CYP2C19 DEL 1.3 N Y DAXX AMP 2.7 N

EGFR AMP 2.6 N Y ELN AMP 2.6 N

ERCC4 AMP,DEL 2.6,1.5 N Y EZH2 DEL 1.5 N

FAT3 DEL 1.5 N Y FBXW7 DEL 1.3 N

FLT1 NORM,DEL 1.9,1.3 N Y FLT3 DEL,NORM 1.4,1.9 N

GABRB3 DEL 1.2 N Y GATA3 AMP 2.9 N

GNAS AMP 2.6 N Y HEATR7B2 DEL 1.4 N

HNF1B AMP 2.7 N Y HRAS AMP 2.7 N

IDH2 AMP 3.9 N Y IGF1R AMP,NORM 3.9,2.4 N

ITGA4 AMP,DEL 3.4,1.4 N Y JAK3 AMP 2.8 N

LPHN3 DEL 1.3 N Y LRP1B DEL 1.3 N

MAP3K1 DEL 1.4 N Y MEN1 AMP 2.7 N

NCOR1 DEL 1.5 N Y NF1 DEL 1.5 N

NOTCH4 AMP 2.7 N Y NPM1 DEL 1.4 N

PAPPA2 AMP,NORM 3.3,1.8 N Y PBRM1 AMP,NORM 2.9,1.5 N

PDGFRA DEL,NORM 1.3,2 N Y PDGFRB AMP 2.8 N

PIKFYVE DEL 1.4 N Y PKHD1 AMP,DEL 2.7,1.3 N

PPP2R1A AMP 2.8 N Y PPP2R4 AMP 3.1 N

PTEN DEL 1.3 N Y PTK2 DEL 1.1 N

RET AMP,DEL 2.9,1.3 N Y RIMS2 NORM,DEL 21.1 N

SMARCA4 AMP 2.8 N Y SMARCB1 AMP 2.8 N

STK11 AMP 2.8 N Y SYK AMP 3.1 N

TGFB1 AMP 2.8 N Y TOP1 AMP,NORM 2.5,1.5 N

TSC2 AMP 2.6 N Y TSHR AMP,DEL 2.6,1.3 N

WT1 AMP 2.7 N Y ZNF238 DEL 1.3 N

**CLIA** **Result**

**Gene** **Result** **Codons** **Amino** **Acid** **Exon** **SAccession** **MAccession** **Comments** **Gene** **Result** **Codons** **Amino** **Acid** **Exon** **SAccession** **MAccession** **Comments**

**Patient** **6**

**T200** **Mutations**

**Freq**

**Gene** **HGNC_AAS** **Protein** **Codons** **Amino** **Position** **Acids** **Exon** **Allele** **Confidence** **Coverage** **Nucleotide** **Position** **dbSNP** **Condel** **Uploaded** **Variation** **P**

APC APC_K1363E 1363 Aaa/Gaa K/E 16/16 21.69% medium 590 4087 NA neutral(0.466) 5_112175378_A/G b

CSF1R CSF1R_A527G 527 gCc/gGc A/G 11/22 18.59% low 597 1580 NA neutral(0.053) 5_149447824_C/G b

HNF1B HNF1B_N357K 357 aaC/aaG N/K 5/9 18.5% low 508 1071 NA neutral(0.298) 17_36070646_C/G b

**T200** **CNA**

**Gene** **Copy** **#** **Alteration** **Estimate** **Copy** **#** **SkipReporting?** **CGMB** **Sent?** **Gene** **Copy** **#** **Alteration** **CN** **SkipReporting?**

CDK4 **H.AMP** 31.2 N Y ACVR1B AMP,DEL 3.1,1.4 N

ARAF NORM,DEL 1.7,1.3 N Y ERBB3 DEL 1.4 N

FLG DEL,NORM 1.4,1.8 N Y FLT1 DEL,NORM 1.4,1.8 N

GABRB3 DEL 1.5 N Y GATA1 DEL 1.3 N

HNF1A DEL 1.4 N Y HRAS DEL 1.3 N

KRAS DEL 1.5 N Y LRP1 DEL 1.4 N

MLL2 DEL 1.5 N Y NAV3 AMP,NORM 3.4,1.9 N

NRAS DEL 1.4 N Y PTPN11 NORM,DEL 1.9,1.4 N

RNF213 DEL,NORM 1.5,1.9 N Y SYNE1 AMP 2.9 N

**CLIA** **Result**

**Gene** **Result** **Codons** **Amino** **Acid** **Exon** **SAccession** **MAccession** **Comments** **Gene** **Result** **Codons**

**CMS46**

APC Variant 1363 K/E S-12-066635 M-13-011173

MET

Variant Prob. Germline

Polymorphism

375 N/S

ﬁle:///Users/nsomaiah/Desktop/liposarcoma/T200_FO_hong_manuscript/IPCT%20T200%20Results%20Full_Internal.html Page 3 of 4

S-12-066635 M-13-011173

**Patient** **7**

**T200** **Mutations**

**Freq**

**Gene** **HGNC_AAS** **Protein** **Codons** **Amino** **Position** **Acids** **Exon** **Allele** **Confidence** **Coverage** **Nucleotide** **Position** **dbSNP** **Condel** **Uploaded** **Variation** **P**

EGFR EGFR_R108K 108 aGa/aAa R/K 3/28 9.06% low 1126 323 NA deleterious(0.945) 7_55211080_G/A p

FLG FLG_D2936G 2936 gAc/gGc D/G 3/3 1.62% low 739 8807 rs80221306 - 1_152278555_A/G p

**Gene** **HGNC_AAS** **Protein** **Position** **Codons** **Amino** **Acids** **Exon** **Allele** **Freq** **Confidence** **Coverage** **Nucleotide** **Position** **dbSNP** **Condel** **Uploaded** **Variation** **P**

EGFR EGFR_R108K 108 aGa/aAa R/K 3/28 9.39% low 841 323 NA deleterious(0.945) 7_55211080_C/T p

**T200** **CNA**

**Gene** **Copy** **#** **Alteration** **Estimate** **Copy** **#** **SkipReporting?** **CGMB** **Sent?** **Gene** **Copy** **#** **Alteration** **CN** **SkipReporting?**

CDK4 **H.AMP** 8.9 Y N EGFR AMP 2.5 Y

MTOR AMP 2.9 Y N RB1 NORM,AMP 2,2.8,2.2 Y

**S-Accession:S-13-070029** **M-Accession:M-13-014566** **IPCT** **SampleID:** **IPCT-CH-3316-5225-A-TU-B01** ***Flowcell:*** ***53***

**Gene** **Copy** **#** **Alteration** **Estimate** **Copy** **#** **SkipReporting?** **CGMB** **Sent?** **Gene** **Copy** **#** **Alteration** **CN** **SkipReporting?**

9-Mar **H.AMP** 8.4 Y N AGAP2 **H.AMP** 8.4 Y

CPM **H.AMP** 9.9,20.3 Y N FRS2 **H.AMP** 14 Y

LYZ **H.AMP** 15.4 Y N MDM2 **H.AMP** 21.4 Y

YEATS4 **H.AMP** 14 Y N YEATS4 **H.AMP** 15.4 Y

FBRS AMP 2.5 Y N FBRS AMP 2.7 Y

MTOR-AS1 AMP 2.9 Y N PRR14 AMP 2.5 Y

SMC3 NORM,AMP 2.2,2.9,1.9 Y N SPEN AMP,NORM 2.9,2.1 Y

**CLIA** **Result**

**Gene** **Result** **Codons** **Amino** **Acid** **Exon** **SAccession** **MAccession** **Comments** **Gene** **Result** **Codons** **Amino** **Acid** **Exon** **SAccession** **MAccession** **Comments**

ﬁle:///Users/nsomaiah/Desktop/liposarcoma/T200_FO_hong_manuscript/IPCT%20T200%20Results%20Full_Internal.html Page 4 of 4
